# Is Modulation of Oxidative Stress an Answer? The State of the Art of Redox Therapeutic Actions in Neurodegenerative Diseases

**DOI:** 10.1155/2016/7909380

**Published:** 2015-12-31

**Authors:** Valerio Chiurchiù, Antonio Orlacchio, Mauro Maccarrone

**Affiliations:** ^1^School of Medicine and Center of Integrated Research, Campus Bio-Medico University of Rome, Rome, Italy; ^2^European Center for Brain Research (CERC), Laboratory of Neurochemistry of Lipids, IRCCS Santa Lucia Foundation, Rome, Italy; ^3^European Center for Brain Research (CERC), Laboratory of Neurogenetics, IRCCS Santa Lucia Foundation, Rome, Italy; ^4^Department of System Medicine, University of Rome “Tor Vergata”, Rome, Italy

## Abstract

The central nervous system is particularly sensitive to oxidative stress due to many reasons, including its high oxygen consumption even under basal conditions, high production of reactive oxygen and nitrogen species from specific neurochemical reactions, and the increased deposition of metal ions in the brain with aging. For this reason, along with inflammation, oxidative stress seems to be one of the main inducers of neurodegeneration, causing excitotoxicity, neuronal loss, and axonal damage, ultimately being now considered a key element in the onset and progression of several neurodegenerative diseases, including Alzheimer's disease, Parkinson's disease, amyotrophic lateral sclerosis, multiple sclerosis, and hereditary spastic paraplegia. Thus, the present paper reviews the role of oxidative stress and of its mechanistic insights underlying the pathogenesis of these neurodegenerative diseases, with particular focus on current studies on its modulation as a potential and promising therapeutic strategy.

## 1. The Role of Oxidative Stress in Neurodegeneration

Although molecular oxygen (O_2_) is crucial for life of most organisms, it is not totally innocuous. The deleterious effects of O_2_ are thought to result from its univalent metabolic reduction that leads to the formation of chemically reactive and toxic species, known as reactive oxygen species (ROS). These include molecules that contain oxygen-centered free radicals, such as the superoxide radical anion (^•^O_2_^−^), the hydroxyl radical (HO^•^), hydroperoxyl radical (HO_2_^•^), and peroxyl radicals (ROO^•^), as well as nonradical derivatives of O_2_ like hydrogen peroxide (H_2_O_2_), hypochlorous acid (HOCl), and peroxynitrite (ONOO^−^) [1–4]. Several sources, either exogenous or endogenous, contribute to intracellular ROS formation. Exogenous sources include radiation, atmospheric pollutants, and chemicals [5]. Endogenously, ROS originate mainly from mitochondria, when ^•^O_2_^−^ is formed by electrons leaking between complexes I and III of the electron-transport chain [6] and through NADPH oxidase, an enzyme that uses NADPH to reduce O_2_, thus generating large amounts of ^•^O_2_^−^ on the membrane surface as a toxic agent during elimination of pathogens [7]. The reactivity of NO^•^ with ROS leads to the formation of many other reactive species, termed reactive nitrogen species (RNS), which include the key effector molecule ONOO^−^, but also other species such as nitrogen dioxide (NO_2_), dinitrogen trioxide (N_2_O_3_), and dinitrogen tetroxide (N_2_O_4_) [8]. In mammals, NO^•^ is an essential biological molecule mostly generated by a family of specific NO synthase (NOS) isozymes: endothelial (eNOS), neuronal (nNOS), and inducible (iNOS) isozymes [9]. However, NO^•^ can be produced also by other redox enzymes such as xanthine oxidase or nonenzymatically by guanidine-substitute L-arginine analogs in the presence of NADPH [10, 11]. Not surprisingly, the major producers of ROS and RNS are indeed immune cells and specifically phagocytic cells, either resident cells in the brain (i.e., microglia) or infiltrated leukocytes, due to their elevated expression of NADPH oxidase, iNOS, and xanthine oxidase [12, 13]. However, cells are equipped with enzymatic and nonenzymatic antioxidant systems to eliminate ROS and RNS, thus maintaining redox homeostasis. Antioxidants include naturally occurring molecules of high or low molecular weight, as well as nutritional antioxidants, whose action is strictly linked to their bioavailability. Naturally occurring antioxidants are mainly enzymes such as superoxide dismutase (SOD), catalase, glutathione peroxidase/reductase (GPx/GR), and peroxiredoxin or molecules like glutathione (GSH), uric acid, pyruvate, amino acids, transferrin, ferritin, and caeruloplasmin. On the other hand, nutritional antioxidants include lipid-soluble antioxidants (*α*-tocopherol, carotenoids, quinones, and some polyphenols) and water-soluble antioxidants (ascorbic acid and some other polyphenols) [2]. Oxidative stress is strictly dependent on the balance between the rate of radicals production and that of their clearance. This overall balance seems to be under the regulation of transcription factor nuclear factor-E2-related factor (Nrf2), which is indeed a central component of cellular defense against oxidative stress [14]. Intriguingly, a very recent discovery reported the presence of a specific protein, termed negative regulator of ROS, that is, NRROS, which is capable of regulating the production of ROS by modulating their generation from phagocytes during inflammatory responses [15]. In particular, this regulator, which is localized in the endoplasmic reticulum, directly interacts with the membrane-bound subunit gp91(phox) of the NADPH oxidase complex and facilitates the degradation of NOX1 and NOX2 proteins, thereby modulating ROS production.

**Figure 1:**
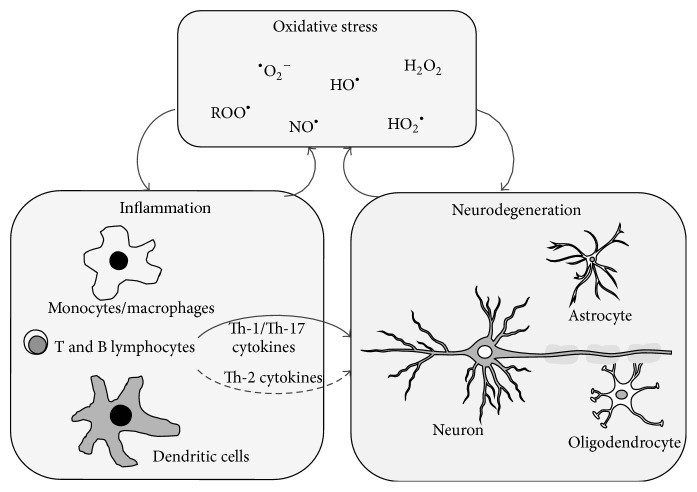
Cross talk between oxidative stress, inflammation, and neurodegeneration. The different reactive species are produced by several cell types, either by resident brain cells (i.e., glia) or by infiltrated leukocytes (i.e., monocytes/macrophages and dendritic cells), and affect both inflammatory processes, by enhancing cytokine release from proinflammatory T cells, and neurodegeneration, by inducing neuronal cell death and axonal loss. ^•^O_2_^−^: superoxide radical anion; HO^•^: hydroxyl radical; ROO^•^: peroxyl radical; HO_2_^•^: hydroperoxyl radical; H_2_O_2_: hydrogen peroxide; NO^•^: nitric oxide; Th: T-helper.

The central nervous system (CNS) is particularly sensitive to oxidative stress and this is due to several reasons. One reason is its high consumption of O_2_ (the brain can metabolize up to ~4 × 10^21^ molecules of glucose per minute.) A second reason is the high production of ROS and RNS, which originate from specific neurochemical reactions (e.g., dopamine oxidation), in addition to the sources discussed previously. A third reason is the increasing deposition of metal ions in the brain with aging, catalyzing the production of increasing levels of ROS and RNS [2]. Another reason is the relatively high abundance of lipids within the CNS (i.e., myelin), which are particularly sensitive to oxidation. For instance, HO_2_^•^ is particularly relevant for the* in vivo* lipid peroxidation and it acts via two different pathways: one that is lipid peroxides independent and the other one that is lipid peroxides dependent [4], leading to the formation of several secondary breakdown products including epoxides and saturated and unsaturated aldehydes such as malondialdehyde (MDA) and 4-hydroxynonenal (HNE), cyclopentenones (i.e., cyclopentenone isoprostanes), and nitro-fatty acids (NO_2_-FAs). Accumulated evidence indicates that oxidative stress plays a major role in the pathogenesis of several neurodegenerative diseases, including Alzheimer's disease (AD), amyotrophic lateral sclerosis (ALS), Parkinson's disease (PD), multiple sclerosis (MS), and hereditary spastic paraplegia (HSP). High levels of ROS and RNS are consistently generated by infiltrating monocytes/macrophages and activated microglia and have been implicated as mediators of neurodegeneration and axonal damage typical of these disorders [16] (Figure 1). Mitochondrial dysfunction in these cells is likely to be one of the causes of such alteration of oxidative metabolism. Along with strong and persistent production of these reactive species, also associated with significant upregulation of their producing enzymes (myeloperoxidase, xanthine, and NADPH oxidases), increases in lipid and DNA oxidation products (i.e., OH8dG, 8-hydroxydeoxyguanosine) have been also reported. Free radicals can also activate certain transcription factors, like NF-*κ*B, which upregulate the expression of many genes involved in neurodegenerative diseases, including proinflammatory cytokines and vascular adhesion molecules. Additionally, redox reactions are involved in the activity of matrix metalloproteinases (MMPs), which are important to cell trafficking into the CNS [17]. Interestingly, along with increased ROS and RNS, direct examination of brain tissues from patients affected by neurodegenerative diseases also revealed a weakened cellular antioxidant defense, especially due to impairment and/or decrease of relevant antioxidants such as superoxide dismutase, catalase, glutathione/glutathione peroxidase, *α*-tocopherol, and uric acid [18]. Indeed, lower levels of antioxidants may promote increased activity of lipoxygenase (that catalyzes one branch of the arachidonate cascade), thereby increasing the immunoinflammatory processes within the brain. In line with this, excessive ROS can stimulate T-cell activity through the arachidonate cascade or can produce direct/indirect damage to the blood brain barrier (BBB) or to neurons [19].

## 2. Alzheimer's Disease

Alzheimer's disease (AD) is probably the most common neurodegenerative disease, accounting for 60% to 70% of cases of dementia with nearly 44 million affected people worldwide, and although its etiology is still unclear, it is characterized by the presence of brain amyloid plaques and neurofibrillary tangles whose accumulation ultimately leads to extensive neuronal loss and progressive decline of cognitive function [2, 39, 40]. They are deposits of proteins distributed throughout the brain of AD patients, particularly in the entorhinal cortex, hippocampus, and temporal, frontal, and inferior parietal lobes. Amyloid plaques are primarily composed of aggregates of *β*-amyloid (A*β*), as well as other protein aggregates (e.g., hyperphosphorylated Tau, ubiquitin, and presenilins 1 and 2), whereas neurofibrillary tangles are aggregates of hyperphosphorylated Tau protein [41]. The production of ROS and its involvement in AD pathogenesis are supported by the significant amount of lipid peroxidation detected in the brain of AD patients, as well as by the increased levels of HNE found postmortem in their cerebrospinal fluid (CSF) [42]. Further, *β*-amyloid-induced damage promotes the generation of ROS, contributing to cell death and neurodegeneration, and induces also glial recruitment and activation, thus triggering local inflammation. Further, oxidative stress promotes abortive cell cycle reentry and hence apoptosis of nerve cells of the adult brain and gene duplication without cell division, leading to aneuploidy and DNA damage [39]. In addition, oxidative stress can damage DNA, leading to strand breaks and large deletions, and can affect various enzymatic and mitogenic pathways. Interestingly, oxidative stress has been shown to decrease neurogenesis in the adult brain, thus limiting its neurodegenerative capacity [43–45]. For the treatment of AD, the current therapy involves drugs that are only able to reduce symptoms or delay disease progression such as acetylcholinesterase inhibitors and those targeting the glutamatergic system. Concerning redox homeostasis, several antioxidant strategies are under study and aim not only at reducing the deleterious activities of ROS, but also at promoting the regenerative capacity of the adult brain [46] (Table 1). These drugs have been experimented in rodent models of AD and include garlic extracts, curcumin, melatonin, resveratrol,* Gingko biloba* extracts, green tea, and vitamin C. Although the clinical value of these antioxidants for the prevention of AD is often elusive, some of these compounds can be recommended based upon epidemiological evidence and already known benefits for prevention of other pathologies [47, 48]. Yet, further long-term studies can be recommended to better understand their mode of action. Vitamin E supplementation in moderately severe AD is to date the most promising approach, although its efficacy is fairly limited and does not apply to all AD patients. The beneficial effect of vitamin E is mainly exerted against peroxidation of membrane lipids of neurons. Several drugs with vitamin E are in use (e.g.,* Sursum*,* Ephynal,* and* Rigentex*) and are orally administered twice a day. Yet, their therapeutic efficacy has not been thoroughly investigated and lack of data reveals the limitations of general antioxidant therapies, whereby they simply lower oxidative stress rather than interfering with the molecular mechanisms underlying disease pathogenesis [47]. In order to design more effective antioxidant therapies against AD, the multiple contributing factors that foster the clinical manifestations of this neurodegenerative disease should be unraveled, particularly in relation to their effects on adult neurogenesis and on synaptic communication. A more sophisticated redox approach involves the interaction between heavy metals and A*β*. For instance, ionic zinc and copper are able to accelerate the aggregation of A*β* and to promote its neurotoxic redox activity by induction of oxidative cross-linking of the peptide into stable oligomers [49]. Therefore, small molecules targeting these interactions are currently under clinical trials and hold promise as disease-modifying agents for AD. These novel drugs are referred to as “metal protein attenuating compounds” (MPAC) [50]. These are different from classical metal chelators, inasmuch as they bear a relatively low affinity for metals and are able to cross the BBB. Furthermore, MPAC stabilize metal homeostasis and interaction with proteins, rather than binding and eliminating metals from tissues. For instance, clioquinol is a zinc/copper ionophore that facilitates the clearance of A*β* aggregates in the cortex of animal models of AD. Its ionophoric properties liberate copper and zinc ions trapped within amyloid plaques, facilitating the reuptake of these essential metal ions into cells and hence promoting memory functions such as long-term potentiation [20]. Oral treatment with this MPAC has been shown to have striking effects in transgenic mouse models of AD, markedly improving learning and memory within days, paralleled by a significant reduction of A*β* content [2]. Other MPAC molecules are being tested in preclinical murine models of AD with the aim of assessing their effect on memory loss and evaluating therapeutic efficacy and toxicity* in vivo*.

## 3. Parkinson's Disease

Parkinson's disease (PD) is the second most common neurodegenerative disorder, affecting an estimated 10 million people worldwide, which produces muscular rigidity, bradykinesia, tremor of resting limbs, and loss of postural balance. The basic neuropathology of PD involves degeneration of pigmented neurons in substantia nigra, resulting in depletion of striatal dopamine (DA) and its metabolites. The pathological hallmarks of PD are large cytoplasmic inclusions called Lewy bodies, which occur predominantly in the melanin-containing neurons of substantia nigra pars compacta (SNpc), and contain aggregates of *α*-synuclein. Another gene encoding a protein termed parkin is involved in autosomal recessive Parkinsonism. Parkin is one member of the family of ubiquitin ligases and may be involved in normal turnover of *α*-synuclein. Although the exact cause of PD is still obscure, both environmental and genetic factors have been implicated in its pathogenesis [21]. Recent evidence points toward a putative role of mitochondrial dysfunction and oxidative stress as well as prooxidant environmental toxicants in the pathogenesis of PD [30, 31], as demonstrated in postmortem brains from PD patients. Apparently, there is a specific chemical fingerprint indicative of the damaging oxidative events, that is, higher levels of cholesterol hydroperoxide, MDA, HNE, and OH8dG. One of the suggested causes of oxidative stress in the SNpc is the production of ROS during normal DA metabolism. In human SNpc, the oxidation products of DA (mainly 6-hydroxydopamine) may polymerize to form neuromelanin, which may also be toxic by inducing apoptosis [32]. Furthermore, postmortem studies revealed reduced levels of GSH and increased levels of GSSG in the SNpc. This could be a critical primary event that weakens or abrogates the natural antioxidant defense of the cell, thereby triggering degeneration of the nigral neurons and causing PD [51]. Since dysregulation of metal ion homeostasis is a potential catalyst to further production of reactive species, the highly oxidative environment for DA interaction with *α*-synuclein, and the resulting oxidant-mediated toxicity and protein aggregation, is one of the most likely underlying mechanisms for PD. Thus, the destruction of neuronal cells occurs as a result of self-propagating reactions that involve DA, *α*-synuclein, and redox-active metals [52]. As for AD, also for PD no cures are available yet. However, pharmacological treatment and surgery could help with symptom relief. The most commonly used drugs to treat motor symptoms are* L*-DOPA, a precursor of dopamine, which is usually used in combination with a DOPA decarboxylase inhibitor and a catechol-*O*-methyltransferase inhibitor; dopamine agonists; and monoamine oxidase inhibitors. These enzymes are all involved in the chemical inactivation of several neurotransmitters, including dopamine. In the early stages of the disease, the treatment aims at controlling both symptoms and side effects caused by dopaminergic enhanced activity, but when the disease gets more severe surgery can be useful. However, in the last stages of PD, palliative care seems to be the only alternative to improve the quality of life [53]. Over the last decade, neuroprotective approaches for PD have been tried with the aim of slowing the rate of disease progression by decreasing oxidative stress (Table 1). There has been much interest in the use of supplemental vitamin E, which seems to inhibit cell death of neuronal cells of SNpc. Regular consumption of vitamin E-rich foods may have the potential to decrease the risk or delay the onset of PD. Even *β*-carotene seems to reduce the risk of PD onset, although no studies to prove its efficacy are available yet. On the other hand, intake of vitamin C and flavonoids did not show any significant beneficial effect in either prevention or treatment of PD. Overall, high intake of dietary antioxidant supplements (mainly vitamin E) might protect against the occurrence of PD rather than treating its symptoms [54, 55]. The use of melatonin and *α*-lipoic acid has also been investigated for PD treatment, but their effects, though promising, have not been fully characterized [56]. The antioxidant with the most efficacious therapeutic potential is coenzyme Q10. Indeed, several clinical trials based on coenzyme Q10 have been undertaken, showing significant beneficial effects on motor functions [22, 23, 57]. Currently, a phase III clinical trial is ongoing and is based on combinations of coenzyme Q10 and vitamin E or creatine, with the aim of evaluating the effective dosage according to the disease stage. Initial results seem to document additive neuroprotective effects in terms of significant reduction of DA depletion in the striatum and loss of tyrosine hydroxylase neurons in the SNpc, as well as reduction in lipid peroxidation and pathologic accumulation of *α*-synuclein in the same SNpc neurons [26]. Further, a double blind phase I/IIa clinical study proved the safety and tolerability of intranasal GSH administration to early and untreated patients, although pharmacokinetic and dose-finding investigations still need to be verified [27]. As already described for AD, another potential therapeutic strategy lies in the modulation of heavy metals, whereby the control of their bioavailability could prevent not only the increase of oxidative stress through metalloredox reactions, but also their interaction with other proteins like *α*-synuclein. Thus, MPAC have been also tested in PD [28] and clioquinol has been reported to reduce cell death of substantia nigra neurons by 50% [29]. Of note, the inhibition of monoamine oxidase isoforms reduces the formation of dopamine-derived peroxides and the subsequent generation of reactive species and of the overall oxidative stress of substantia nigra.

## 4. Amyotrophic Lateral Sclerosis

Amyotrophic lateral sclerosis (ALS) is a fatal neurodegenerative disease characterized by the death of both upper and lower motor neurons of the brain, brain stem, and spinal cord, overall leading to progressive weakness and atrophy of skeletal muscles [2, 33]. Approximately 10% of the cases are inherited in an autosomal dominant manner, and 1/5 of these familial ALS patients carries mutations in the Cu/Zn-SOD (SOD-1) gene, suggesting involvement of ROS in disease pathogenesis [34]. The toxicity of mutant SOD-1 seems to be due to gain of function of this enzyme, whereby its catalytic activity is enhanced with abnormal substrates like ONOO^−^, thus sustaining nitration of tyrosine and subsequent oxidative stress. This may also be related to impaired ability of mutant SOD-1 to bind zinc, because* in vivo* the mutant enzyme is likely to denature more quickly than the normal form, releasing zinc and copper ions. Oxidative stress may also be involved in misfolding of mutant SOD-1, to yield abnormal protein aggregates that can be found as early as in 1-month-old SOD-1-mutant mice [58]. Also, the disorganization of intermediate filaments could be due to mutant SOD-1-induced toxicity, as these cytoskeletal proteins are vulnerable to oxidative damage [59]. In addition, protein carbonyl and nitrotyrosine modifications, which are indexes of protein oxidation, were found to be elevated in the majority of patients with sporadic ALS, suggesting that oxidative stress may indeed be involved in all types of ALS. Other mechanisms that have been implied in ALS, such as excitotoxicity and defective axonal transport, may be consequences of oxidative stress [60]. What remains as yet unclear is whether this increased redox stress is a primary defect or a secondary consequence of the disease. An interesting study has demonstrated that SOD-1-mutant ALS transgenic mice activate cellular Nox2 activity and subsequent ^•^O_2_^−^ production in spinal cord microglia, through disruption of the redox-sensitive regulation of Rac1-dependent Nox activation [61]. Of note, Nox enzymes control relevant proinflammatory signaling pathways involved in the progression of ALS, such as those mediated by IL-1*β* and TNF-*α* via redox-dependent activation of NF-*κ*B. Finally, marked elevation of 2-thiobarbituric reactive substances in plasma (i.e., MDA) was found both in mutant SOD-1 mice and in patients with sporadic ALS [62, 63]. However, plasma concentrations of antioxidants like *α*-tocopherol, *β*-carotene, ubiquinol-10, and GSH, as well as SOD activity in red blood cells, were not significantly different between ALS patients and healthy subjects [63]. Even though we still lack a definitive cure for ALS, the Food and Drug Administration (FDA) has already approved the first molecule to treat the disease, that is, riluzole. This drug is thought to reduce the motoneuron-associated damage by affecting the release of glutamate. Treatment with riluzole of ALS patients in clinical trials only elicited a three-month improvement of survival rate, with the subjects needing constant monitoring of liver damage and other side effects [64]. Nonetheless, this first attempt of a specifically aimed therapy nurtures the hope that the clinical course of ALS might be managed by new kinds of treatments or combinations of new drugs. Moreover, several antioxidant molecules have been tested as putative therapeutic agents in the treatment of ALS (Table 1). Such compounds include NAC and N-acetylmethionine (NAM), vitamins C and E, resveratrol, dithiotreitol (or any of its isomers), and dithioeritrol. However, although such antioxidant agents never caused noxious effects, all of them failed in eliciting any kind of significant effect on patients' survival rate [65]. To date, two antioxidant compounds have been used in clinical trials. The first one is manganese-metalloporphyrin, which was able to extend survival rate in murine models [66]. Metalloporphyrins are compounds made by a tetrapyrrole ring that coordinates a central metal atom. Two phase I trials, conducted to assess possible drug-associated toxicity, showed very good tolerance of this compound even at fairly high doses (up to 2 mg/kg/day), with ALS patients showing an excellent pharmacokinetic profile. The second antioxidant drug under investigation is KNS-760704, a pramipexole enantiomer, usually used for treatment of PD patients, which acts as a dopamine receptor agonist [67]. This compound possesses ROS-scavenging activity and has been proven to extend the lifespan of ALS animal models. Moreover, a recent phase II clinical trial also reported that KNS-76074 is able to exert protective action against oxidative stress-associated neurotoxic damage, which led this compound to be tested in phase III trials in order to evaluate its efficacy and tolerability. New therapeutic approaches are currently being developed with the aim of blocking the production of SOD-1 mutated forms, a strategy that could likely ameliorate the clinical course of patients affected by the familial form of ALS. Curiously, a recent study pointed out that the mutant SOD-1 transgenic mice model of ALS is not representative of the human sporadic form, accounting for most failures in experimental or clinical research.

## 5. Multiple Sclerosis

Multiple sclerosis (MS) is a chronic inflammatory, progressive, and degenerative disorder of autoimmune origin characterized by intermittent episodes of demyelination and axonal loss or damage in the CNS. Although its etiology has not been fully elucidated yet, it is likely that both genetic and environmental components play a crucial role in disease onset and progression, and it is now well recognized that immunological mechanisms are the initial trigger [2, 16, 37]. MS is classified into four independent subtypes or forms: relapsing-remitting (RR), primary progressive (PP), secondary progressive (SP), and progressive relapsing (PR); the former is the most prevalent form and accounts for approximately 85% of all cases [38]. Interestingly, its pathogenesis and pathophysiology have been extensively studied, especially on experimental autoimmune encephalomyelitis (EAE) mouse model, and are thought to be due to disruption either of the immune system or of the myelin-producing cells. As a matter of fact, the hallmarks of MS are inflammation and neurodegeneration, where, upon damage of the BBB, massive infiltration of highly proinflammatory and autoreactive leukocytes occurs (especially T-helper 1 and T-helper 17 cells), causing demyelination as well as oligodendrocyte death, axon damage, and even neuronal loss [68]. These autoimmune processes are paralleled by continuous activation of resident macrophages/microglia that potentiate the inflammatory response by producing proinflammatory cytokines and chemokines, as well as reactive oxidants [69]. The autoimmune and inflammatory hypothesis dominated the MS research field for almost 50 years until the early 2000s; however, whether inflammatory demyelination is primary or secondary in the disease process is yet unclear. Indeed, over the last decade, the concept of a neurodegenerative and microglia-centered view has been gaining increasing attention. In this view, MS might be primarily a neurodegenerative disease with secondary inflammatory demyelination, whose trigger starts in the brain and the progress of which is modified and amplified by inflammation [70, 71]. Accumulated evidence indicates that oxidative stress plays a major role in the pathogenesis of MS. ROS and RNS are mainly generated in excess by activated microglia and have been implicated as mediators of demyelination and axonal damage typical of MS [72]. In addition, free radicals can activate certain transcription factors, like NF-*κ*B, which upregulate the expression of many genes involved in human MS and EAE, including TNF-a, iNOS, ICAM-1, and VCAM-1 [73]. Additionally, redox reactions are involved in the activity of matrix metalloproteinases (MMPs), which are important to T-cell trafficking into the CNS [74]. Several studies have found evidence of lipid peroxidation in the CSF and plasma of MS patients, with higher concentration of isoprostanes and MDA. Further, a weakened cellular antioxidant defense, especially due to impairment of SOD, GR, and GPx, as well as elevated levels of GSSG and reduced vitamin E : lipid ratio, was found in red blood cells of these subjects [75]. Moreover, direct examination of MS plaques revealed an increase in free radical activity and decreased levels of relevant antioxidants like GSH, a-tocopherol, and uric acid [18]. Furthermore, activated mononuclear cells of MS patients produce high amounts of ROS and NO^•^, and oxidative damage to DNA (mitochondrial DNA included) develops in association with inflammation in chronic active plaques [76]. Another study documented that oxidative damage of CNS was provoked by the release of iron from injured cells and by low levels of enzymatic and nonenzymatic antioxidants (particularly ubiquinone and vitamin E) in plasma and lymphocytes of MS patients [77]. The CNS damage induced by low levels of antioxidants or high levels of ROS might be caused by the fact that lower levels of antioxidants may promote increased activity of lipoxygenase (that catalyzes one branch of the arachidonate cascade) [78], thereby increasing the immunoinflammatory processes within the brain, and by the evidence that excessive ROS can stimulate T-cell activity via the arachidonate cascade, or they can produce direct/indirect damage to the BBB or to myelin [79]. As yet, a final treatment for MS has not been found, and no therapy has been developed to date for its progressive forms, even though RR-MS can count on several disease-modifying treatments (DMTs). Besides methylprednisolone, a corticosteroid used immediately after the diagnosis of MS and before any other more specific therapeutic approaches, FDA has approved seven other DMTs which are already in commerce: Interferon-*β*-1a (IFN-*β*-1a), IFN-*β*-1b, Glatiramer acetate, Mitoxantrone, Teriflunomide, Fingolimod, and Natalizumab [16]. Such drugs are all immunomodulatory and aim at halting the pathological immune responses by directly inhibiting cell activation and the release of proinflammatory mediators or by limiting cell transmigration into the CNS; yet they still do not represent a definitive solution to the problem, especially for the 15–20% of MS patients affected by the progressive forms of MS. Given the involvement of ROS and RNS in MS pathogenesis, it is possible that antioxidant compounds could play a pivotal role in the prevention of the free radical-mediated tissue damage as well as inhibiting the early proinflammatory events, such as T-cell activation and CNS infiltration, which would ultimately lead to brain inflammation and neuronal death [16]. Treatment with antioxidant could theoretically prevent the spreading of tissue damage promoting cellular survival, thus ameliorating the disease outcome (Table 1). In this context, the antioxidant compound tirilazad mesylate, a member of lazaroid family known for its peroxyl-radical-scavenger properties and for its ability to reduce iron-catalysed lipid peroxidation, has been proven useful in preventing the onset of acute EAE (the animal model of MS), as well as reducing its severity. Another study showed that the administration of NAC, a molecule particularly efficient in boosting intracellular levels of GSH, is able to hinder the induction of acute EAE [80]. On the other hand, Euk-8, a synthetic salen-manganese complex, could emerge as a key compound in the development of a brand new class of molecules possessing scavenging properties along with superoxide dismutase and catalase abilities. As a matter of fact, repeated injections of Euk-8 upon encephalomyelitis induction in mice were able to delay the onset of EAE symptomatic phase as well as reduce the severity of the clinical phenotype, giving hope to the hypothesis of its possible application in human MS [81]. *α*-Lipoic acid, an antioxidant molecule capable of crossing the BBB, has also been recently reported to suppress inflammation, demyelination, and axonal damage in EAE mice. Its effects are mediated by the reduction of T-cell traffic in the spinal cord, possibly through the inhibition of MMPs activity. Uric acid too has been proven to affect EAE clinical outcome, especially ameliorating neurologic deficits in treated mice, in a mechanism involving its ability to inhibit iNOS along with NO^•^ and ONOO^−^ scavenging properties [82]. However, other authors reported that inhibition of NO^•^ production is instead deleterious in EAE, leaving a wide-open debate on the true efficacy of this compound. Some other encouraging data obtained from EAE mice led the scientific community to theorize that the dietary income of antioxidant, such as vitamin E or selenium, could somehow hinder the progression of MS. Of note, despite the relative abundance of reports describing the ameliorative effects of restrictive dietary regimens on the clinical outcome of affected patients, we still lack a true and solid body of evidence supporting the actual action of antioxidant in slowing the progression of MS [83]. Natural occurring molecules currently being investigated in phase I and II trials include polyphenols (especially* Gingko biloba* extracts), essential fatty acids/*α*-lipoic acid, and vitamin E/selenium [84]. The results of these promising studies will lay the foundation for phase III trials, which will be pivotal in establishing the long-term efficacy of antioxidant therapies on MS. An ongoing phase I-II trial is recruiting patients in order to assay the effects of idebenone, a synthetic compound chemically related to coenzyme Q10. Currently gathered data suggest that idebenone could be able to stop demyelination and neuronal death. Another study is also investigating the efficacy of *ω*-3 PUFAs included in fish oil such as eicosapentaenoic acid (EPA) and docosahexaenoic acid (DHA) as anti-inflammatory, antioxidant, and neuroprotective agents [24]. MS patients treated with fish oil (4 g/die) showed a significant reduction of the levels of proinflammatory cytokines and NO^•^ catabolites, but no variation in the serum levels of lipoperoxides or the number of relapses per year [25]. The results coming from such studies are laying the foundations for a phase III clinical trial that should give information about the long-term efficacy of this strategy on the number of relapses. The use of antioxidants, even in combination with conventional immunomodulatory therapies, could have synergistic effects on the disease, resulting in a more powerful therapy. Indeed, in order to evaluate the real benefit of antioxidant therapies on MS patients, adequately designed clinical studies will be needed in conjunction with observational investigations that will evaluate on sufficiently large cohorts of patients the long-term effectiveness of this potential treatment. The pivotal role of oxidative stress in MS pathogenesis and the idea that a therapeutic strategy could reside in the control of such a phenomenon is highly supported by the recent approval of the first redox-modulating drug in the treatment of multiple sclerosis. This compound, dimethyl fumarate (DMF), which is a methyl ester of fumaric acid, is the only oral administration DMT to be approved by both FDA and the European Medicines Agency (EMA), with the trade name Tecfidera. This compound was initially used in the treatment of psoriasis, though administered as a different preparation; however, it was later proposed as therapeutic drug for MS due to the immunopathogenic features these two diseases share. Right after administration, the small intestine esterases readily hydrolyze DMF into mono-methyl fumarate (MMF), which is more stable and possesses a 12-hour* in vivo* half-life, higher than its precursor. The mechanism of action of DMF is based on its ability to interfere with the redox-regulating cellular systems and the consequent modulation of intracellular thiols, which in turn boosts GSH levels. In detail, DMF interacts with Kelch-like erythroid cell-derived protein with cap “n” collar homology-associated protein 1 Keap-1 at the level of its critical cysteine residue Cys151 which is covalently adducted, leading to cleavage of this protein and the subsequent translocation and activation of Nrf-2, which in turn triggers several cellular antioxidant pathways, resulting in anti-inflammatory and neuroprotective responses [35]. The detailed action is based on the Nrf-2-induced expression of proteins that regulate intracellular antioxidant systems such as NQO1, heme-oxygenase-1 (HO-1), glutathione S-transferase Mu-1 (GSTM1), Prx1, and Trx, along with a vast number of heat shock proteins (HSPs), thus promoting immune cell survival even in the presence of high ROS and RNS concentrations [36]. Thus, Nrf-2 orchestrates a complex machinery that protects neurons and glial cells against the oxidative stress-induced cell damage. Moreover, a great deal of evidence suggests that DMF is also able to modulate immune responses either through the Nrf-2-dependent inhibition of the redox signals governed by NF-*κ*B and/or skewing Th1/Th2 balance towards Th2 by directly inducing T-cell apoptosis, without however resulting in immunosuppression. DMF has been also shown to protect neural stem/progenitor cells and neurons from oxidative damage through Nrf2-ERK1/2 MAPK pathway [85, 86]. As a consequence, BG-12, a specific oral preparation of DMF, has already been showed in two phase III trials as being able to reduce relapse episodes as well as to delay disease progression in patients affected by RR-MS [87–89]. In these trials, BG-12 was well tolerated, the most common side effects being characterized by redness, gastrointestinal symptoms, and headaches. Another phase III clinic trial is currently investigating BG-12 long-term safety profile with the main aim of using it in the future as a first-line DMT.

## 6. Hereditary Spastic Paraplegia

Hereditary spastic paraplegia (HSP) includes a large and diverse group of genetic disorders whose main feature is progressive spasticity and weakness in the lower limbs, as a result of continuous distal axonopathy caused by defects in the mechanisms that transport proteins and substances along the axons [90, 91]. At least four autosomal dominant HSPs are caused by mutations in genes encoding proteins that are involved in ER morphogenesis and that bear an intramembrane hairpin loop responsible for the curvature of ER membranes and for their reciprocal interactions. These include spastin, the most commonly mutated protein in HSP, atlastin-1, REEP1, and RTN2 [91–93]. To date, 72 different spastic gait disease loci have been identified, and 55 spastic paraplegia genes (SPGs) have already been cloned, most of which play a role in intracellular trafficking [94]. The products of these genes are all implicated in disease onset of many forms of HSP and can be grouped into “functional modules” in which they are part of specific molecular pathways or perform similar functions, including dysfunctional axonal transport, axon development, dysregulation of myelination, and abnormal cellular signaling in protein morphogenesis. Oxidative stress is, in fact, one of these functional modules inasmuch as many of these genes affect mitochondrial function and thus determine increase in ROS and RNS production, [95]. Indeed, several reports suggest that oxidative stress could be strictly involved in the pathogenesis of many forms of HSP. In this context, one of the most studied genes is paraplegin (SPG7), a mitochondrial metalloprotease belonging to the family of ATPases associated with diverse cellular activities (AAA) [96, 97], whose mutations result in mitochondrial dysfunction of muscle tissue and mitochondrial-dependent impairment of axonal transport [98, 99]. HSP fibroblasts of patients affected by SPG7 show reduced complex I activity in mitochondria and an increased sensitivity to oxidative stress [100]. Such dysfunction could directly contribute to neurodegeneration via free radical mechanism by direct ROS production and by a decreased ATP synthesis leading to energy failure. Of note, HSP cells are more sensitive to DNA damage induced by H_2_O_2_ treatment [101], also because abnormal DNA repair is another functional module associated with HSP. Also another form of HSP, that is, SPG13, is caused by mutations in the HSPD1 gene that encodes heat shock protein 60, which is crucial for the folding of several mitochondrial proteins, once again affecting mitochondrial function [102]. Further, abnormal lipid metabolism is another key functional module and is also associated with oxidative stress. For instance, mutations in* DDHD1* and* CYP2U1* genes, which code for two enzymes involved in fatty-acid metabolism, cause alteration of mitochondrial architecture and bioenergetics with increased oxidative stress, accounting for lipid metabolism as a critical HSP pathway with a deleterious impact on mitochondrial bioenergetic function [103]. Overall, these lines of evidence suggest that oxidative stress is likely a crucial biomarker and a novel pathogenic mechanism for these neurodegenerative disorders. No specific treatments are yet available to prevent, slow, or reverse HSP. The available therapies mainly deal with management of symptoms and physical and emotional promotion and include drugs for muscle tone and spasms as well as antidepressants for patients experiencing clinical depression. Physical therapy is also used to restore and maintain the ability to move. The probable implication of mitochondrial dysfunction and oxidative stress in the pathophysiology of HSP set the basis for the development of novel therapeutic strategies focused on organelle dynamics and bioenergetics, as well as ROS scavenging. To date, only one clinical trial is investigating the therapeutic potential of targeting oxidative stress in HSP and is investigating the efficacy of resveratrol in order to decrease the production of oxysterols by reducing the synthesis of cholesterol and/or regulating the production of bile acids and/or enabling neuroprotective action within the motor neuron [ClinicalTrials.gov: NCT02314208]. Of note, one of the challenges to accurately define the HSP pathways and to design efficacious therapies is that many genes have multiple functions and are involved in more than one pathway.

## 7. Conclusions

Due to the harmful role of ROS and RNS in the pathology of neurodegenerative diseases, antioxidants seem to limit the tissue damage induced by these reactive species through inhibition of early or late proinflammatory events or exerting neuroprotective properties. Accordingly, several lines of research considered nutritional interventions with vitamins, micronutrients, and antioxidants to complement conventional treatments; yet only few of them were translated into clinical practice. This is due to multiple reasons, including the large number of reactive species and the multiple routes for their metabolism that simultaneously occur and that interact with each other, the overlapping of common signaling redox pathways in health and disease, and the scarce knowledge of their mechanistic insights* in vivo*. Furthermore, it must be considered that the main problem regarding the treatment of neurodegenerative diseases is embodied by the necessity of developing compounds that are able to cross the BBB, that is, the main obstacle between the CNS milieu and the peripheral bloodstream. The BBB is able to reduce the efficacy of antioxidant drugs as well as several other compounds, which may exert therapeutic action. Consequently, future development of antioxidant therapeutics will undoubtedly depend on a wider knowledge of the BBB-associated transport mechanisms. Yet, targeting specific oxidative stress pathways, rather than the use of dietary antioxidants, seems to represent a novel avenue of research in the management of neurodegenerative diseases, especially after the recent approval of dimethyl fumarate by both the FDA and EMA which acts by enhancing the antioxidant responses, ultimately promoting cytoprotection of neurons and glial cells.

## Figures and Tables

**Table 1:** Current redox clinical investigations for the treatment of neurodegenerative diseases.

Redox therapy	Clinical application
Vitamin E	Alzheimer's disease [20, 21] Parkinson's disease [22, 23] Multiple sclerosis [24, 25]

Polyphenols	Parkinson's disease [22, 23] Multiple sclerosis [24, 25] Hereditary spastic paraplegia [ClinicalTrials.gov: NCT02314208]

Coenzyme Q10	Parkinson's disease [26–29] Multiple sclerosis

MPAC (i.e., clioquinol)	Alzheimer's disease [30–32] Parkinson's disease [33, 34]

*ω*-3 PUFAs	Multiple sclerosis [35, 36]

Metalloporphyrins	Amyotrophic lateral sclerosis [37]

Pramipexole (i.e., KNS-760704)	Amyotrophic lateral sclerosis [38]

MPAC: metal protein attenuating compounds; PUFAs: polyunsaturated fatty acids.

## References

[1] Halliwell B., Gutteridge J. M. (1999). *Free radicals in Biology and Medicine*.

[2] Chiurchiù V., MacCarrone M. (2011). Chronic inflammatory disorders and their redox control: from molecular mechanisms to therapeutic opportunities. *Antioxidants and Redox Signaling*.

[3] Dröge W. (2002). Free radicals in the physiological control of cell function. *Physiological Reviews*.

[4] Aikens J., Dix T. A. (1991). Perhydroxyl radical (HOO) initiated lipid peroxidation: the role of fatty acid hydroperoxides. *The Journal of Biological Chemistry*.

[5] Trachootham D., Lu W., Ogasawara M. A., Valle N. R.-D., Huang P. (2008). Redox regulation of cell survival. *Antioxidants & Redox Signaling*.

[6] Raha S., Robinson B. H. (2000). Mitochondria, oxygen free radicals, disease and ageing. *Trends in Biochemical Sciences*.

[7] Henderson L. M., Chappell J. B. (1996). NADPH oxidase of neutrophils. *Biochimica et Biophysica Acta: Bioenergetics*.

[8] Stamler J. S., Singel D. J., Loscalzo J. (1992). Biochemistry of nitric oxide and its redox-activated forms. *Science*.

[9] Leiper J., Nandi M. (2011). The therapeutic potential of targeting endogenous inhibitors of nitric oxide synthesis. *Nature Reviews Drug Discovery*.

[10] Moroz L. L., Norby S. W., Cruz L., Sweedler J. V., Gillette R., Clarkson R. B. (1998). Non-enzymatic production of nitric oxide (NO) from NO synthase inhibitors. *Biochemical and Biophysical Research Communications*.

[11] Zanotto-Filho A., Schröder R., Moreira J. C. F. (2008). Xanthine oxidase-dependent ROS production mediates vitamin A pro-oxidant effects in cultured sertoli cells. *Free Radical Research*.

[12] Yang Y., Bazhin A. V., Werner J., Karakhanova S. (2013). Reactive oxygen species in the immune system. *International Reviews of Immunology*.

[13] Lugrin J., Rosenblatt-Velin N., Parapanov R., Liaudet L. (2014). The role of oxidative stress during inflammatory processes. *Biological Chemistry*.

[14] Kobayashi M., Yamamoto M. (2005). Molecular mechanisms activating the Nrf2-Keap1 pathway of antioxidant gene regulation. *Antioxidants & Redox Signaling*.

[15] Noubade R., Wong K., Ota N. (2014). NRROS negatively regulates reactive oxygen species during host defence and autoimmunity. *Nature*.

[16] Chiurchiù V. (2014). Novel targets in multiple sclerosis: to oxidative stress and beyond. *Current Topics in Medicinal Chemistry*.

[17] Leppert D., Waubant E., Galardy R., Bunnett N. W., Hauser S. L. (1995). T cell gelatinases mediate basement membrane transmigration in vitro. *Journal of Immunology*.

[18] Langemann H., Kabiersch A., Newcombe J. (1992). Measurement of low-molecular weight antioxidants, uric acid, tyrosine and tryptophan in plaques and white matter from patients with multiple sclerosis. *European Neurology*.

[19] Lehner C., Gehwolf R., Tempfer H. (2011). Oxidative stress and blood-brain barrier dysfunction under particular consideration of matrix metalloproteinases. *Antioxidants & Redox Signaling*.

[20] Robert A., Liu Y., Nguyen M., Meunier B. (2015). Regulation of copper and iron homeostasis by metal chelators: a possible chemotherapy for Alzheimer's disease. *Accounts of Chemical Research*.

[21] Jankovic J. (2008). Parkinson's disease: clinical features and diagnosis. *Journal of Neurology, Neurosurgery and Psychiatry*.

[22] Shults C. W., Oakes D., Kieburtz K. (2002). Effects of coenzyme Q10 in early Parkinson disease: evidence of slowing of the functional decline. *Archives of Neurology*.

[23] Storch A., Jost W. H., Vieregge P. (2007). Randomized, double-blind, placebo-controlled trial on symptomatic effects of coenzyme Q10 in Parkinson disease. *Archives of Neurology*.

[24] Shinto L., Marracci G., Baldauf-Wagner S. (2009). Omega-3 fatty acid supplementation decreases matrix metalloproteinase-9 production in relapsing-remitting multiple sclerosis,. *Prostaglandins Leukotrienes and Essential Fatty Acids*.

[25] Ramirez-Ramirez V., Macias-Islas M. A., Ortiz G. G. (2013). Efficacy of fish oil on serum of TNF*α*, IL-1*β*, and IL-6 oxidative stress markers in multiple sclerosis treated with interferon beta-1b. *Oxidative Medicine and Cellular Longevity*.

[26] Li Z., Wang P., Yu Z. (2015). The effect of creatine and coenzyme q10 combination therapy on mild cognitive impairment in Parkinson's disease. *European Neurology*.

[27] Mischley L. K., Leverenz J. B., Lau R. C. (2015). A randomized, double-blind phase I/IIa study of intranasal glutathione in Parkinson's disease. *Movement Disorders*.

[28] Andersen J. K., Kaur D., Yantiri F. (2003). Genetic or pharmacological iron chelation prevents MPTP-induced neurotoxicity in vivo: a novel therapy for Parkinson's disease. *Neuron*.

[29] Bareggi S. R., Cornelli U. (2012). Clioquinol: review of its mechanisms of action and clinical uses in neurodegenerative disorders. *CNS Neuroscience & Therapeutics*.

[30] Blesa J., Trigo-Damas I., Quiroga-Varela A., Jackson-Lewis V. R. (2015). Oxidative stress and Parkinson’s disease. *Frontiers in Neuroanatomy*.

[31] Pamphlett R. (2014). Uptake of environmental toxicants by the locus ceruleus: a potential trigger for neurodegenerative, demyelinating and psychiatric disorders. *Medical Hypotheses*.

[32] Berman S. B., Hastings T. G. (1999). Dopamine oxidation alters mitochondrial respiration and induces permeability transition in brain mitochondria: implications for Parkinson's disease. *Journal of Neurochemistry*.

[33] Swinnen B., Robberecht W. (2014). The phenotypic variability of amyotrophic lateral sclerosis. *Nature Reviews Neurology*.

[34] Ghadge G. D., Lee J. P., Bindokas V. P. (1997). Mutant superoxide dismutase-1-linked familial amyotrophic lateral sclerosis: molecular mechanisms of neuronal death and protection. *The Journal of Neuroscience*.

[35] Ruggieri S., Tortorella C., Gasperini C. (2014). Pharmacology and clinical efficacy of dimethyl fumarate (BG-12) for treatment of relapsing-remitting multiple sclerosis. *Therapeutics and Clinical Risk Management*.

[36] Fox R. J., Kita M., Cohan S. L. (2014). BG-12 (dimethyl fumarate): a review of mechanism of action, efficacy, and safety. *Current Medical Research and Opinion*.

[37] Noseworthy J. H., Lucchinetti C., Rodriguez M., Weinshenker B. G. (2000). Multiple sclerosis. *The New England Journal of Medicine*.

[38] McDonald W. I., Compston A., Edan G. (2001). Recommended diagnostic criteria for multiple sclerosis: guidelines from the International Panel on the diagnosis of multiple sclerosis. *Annals of Neurology*.

[39] Querfurth H. W., LaFerla F. M. (2010). Alzheimer's disease. *The New England Journal of Medicine*.

[40] Huang Y., Mucke L. (2012). Alzheimer mechanisms and therapeutic strategies. *Cell*.

[41] LaFerla F. M., Green K. N., Oddo S. (2007). Intracellular amyloid-*β* in Alzheimer's disease. *Nature Reviews Neuroscience*.

[42] Di Bona D., Scapagnini G., Candore G. (2010). Immune-inflammatory responses and oxidative stress in Alzheimer's disease: therapeutic implications. *Current Pharmaceutical Design*.

[43] Zhu X., Raina A. K., Lee H.-G., Casadesus G., Smith M. A., Perry G. (2004). Oxidative stress signalling in Alzheimer's disease. *Brain Research*.

[44] von Bernhardi R., Eugenín J. (2012). Alzheimer's disease: redox dysregulation as a common denominator for diverse pathogenic mechanisms. *Antioxidants and Redox Signaling*.

[45] Butterfield D. A., Gnjec A., Poon H. F. (2006). Redox proteomics identification of oxidatively modified brain proteins in inherited Alzheimer's disease: an initial assessment. *Journal of Alzheimer's Disease*.

[46] Lee H. P., Zhu X., Casadesus G. (2010). Antioxidant approaches for the treatment of Alzheimers disease. *Expert Review of Neurotherapeutics*.

[47] Frank B., Gupta S. (2005). A review of antioxidants and Alzheimer's disease. *Annals of Clinical Psychiatry*.

[48] Ferreira M. E. S., de Vasconcelos A. S., da Costa Vilhena T. (2015). Oxidative stress in Alzheimer’s disease: should we keep trying antioxidant therapies?. *Cellular and Molecular Neurobiology*.

[49] Bush A. I., Tanzi R. E. (2008). Therapeutics for Alzheimer's disease based on the metal hypothesis. *Neurotherapeutics*.

[50] Sampson E. L., Jenagaratnam L., McShane R. (2014). Metal protein attenuating compounds for the treatment of Alzheimer's dementia. *The Cochrane Database of Systematic Reviews*.

[51] Gu F., Chauhan V., Chauhan A. (2015). Glutathione redox imbalance in brain disorders. *Current Opinion in Clinical Nutrition and Metabolic Care*.

[52] Carboni E., Lingor P. (2015). Insights on the interaction of alpha-synuclein and metals in the pathophysiology of Parkinson's disease. *Metallomics*.

[53] George J. L., Mok S., Moses D. (2009). Targeting the progression of Parkinson's disease. *Current Neuropharmacology*.

[54] Sutachan J. J., Casas Z., Albarracin S. L. (2012). Cellular and molecular mechanisms of antioxidants in Parkinson's disease. *Nutritional Neuroscience*.

[55] Seidl S. E., Santiago J. A., Bilyk H., Potashkin J. A. (2014). The emerging role of nutrition in Parkinson's disease. *Frontiers in Aging Neuroscience*.

[56] Miller E., Morel A., Saso L., Saluk J. (2015). Melatonin redox activity. Its potential clinical applications in neurodegenerative disorders. *Current Topics in Medicinal Chemistry*.

[57] Yang L., Calingasan N. Y., Wille E. J. (2009). Combination therapy with Coenzyme Q10 and creatine produces additive neuroprotective effects in models of Parkinson's and Huntington's Diseases. *Journal of Neurochemistry*.

[58] Bruijn L. I., Houseweart M. K., Kato S. (1998). Aggregation and motor neuron toxicity of an ALS-linked SOD1 mutant independent from wild-type SOD1. *Science*.

[59] McLean J. R., Smith G. A., Rocha E. M. (2014). ALS-associated peripherin spliced transcripts form distinct protein inclusions that are neuroprotective against oxidative stress. *Experimental Neurology*.

[60] Carter B. J., Anklesaria P., Choi S., Engelhardt J. F. (2009). Redox modifier genes and pathways in amyotrophic lateral sclerosis. *Antioxidants and Redox Signaling*.

[61] Harraz M. M., Marden J. J., Zhou W. (2008). SOD1 mutations disrupt redox-sensitive Rac regulation of NADPH oxidase in a familial ALS model. *Journal of Clinical Investigation*.

[62] Hall E. D., Andrus P. K., Oostveen J. A., Fleck T. J., Gurney M. E. (1998). Relationship of oxygen radical-induced lipid peroxidative damage to disease onset and progression in a transgenic model of familial ALS. *Journal of Neuroscience Research*.

[63] Oteiza P. I., Uchitel O. D., Carrasquedo F., Duborovski A. L., Roma J. C., Fraga C. G. (1997). Evaluation of antioxidants, protein, and lipid oxidation products in blood from sporadic amyotrophic lateral sclerosis patients. *Neurochemical Research*.

[64] Miller R. G., Mitchell J. D., Lyon M., Moore D. H. (2003). Riluzole for amyotrophic lateral sclerosis (ALS)/motor neuron disease (MND). *Amyotrophic Lateral Sclerosi & Other Motor Neuron Disorders*.

[65] Vyth A., Timmer J. G., Bossuyt P. M. M., Louwerse E. S., Vianney De Jong J. M. B. (1996). Survival in patients with amyotrophic lateral sclerosis, treated with an array of antioxidants. *Journal of the Neurological Sciences*.

[66] Crow J. P., Calingasan N. Y., Chen J., Hill J. L., Beal M. F. (2005). Manganese porphyrin given at symptom onset markedly extends survival of ALS mice. *Annals of Neurology*.

[67] Gribkoff V. K., Bozik M. E. (2008). KNS-760704 [(6R)-4,5,6,7-tetrahydro-N6-propyl-2, 6-benzothiazole-diamine dihydrochloride monohydrate] for the treatment of amyotrophic lateral sclerosis. *CNS Neuroscience & Therapeutics*.

[68] Broux B., Stinissen P., Hellings N. (2013). Which immune cells matter? The immunopathogenesis of multiple sclerosis. *Critical Reviews in Immunology*.

[69] Gandhi R., Laroni A., Weiner H. L. (2010). Role of the innate immune system in the pathogenesis of multiple sclerosis. *Journal of Neuroimmunology*.

[70] Trapp B. D., Nave K.-A. (2008). Multiple sclerosis: an immune or neurodegenerative disorder?. *Annual Review of Neuroscience*.

[71] Lassmann H., van Horssen J., Mahad D. (2012). Progressive multiple sclerosis: pathology and pathogenesis. *Nature Reviews Neurology*.

[72] Bö L., Dawson T. M., Wesselingh S. (1994). Induction of nitric oxide synthase in demyelinating regions of multiple sclerosis brains. *Annals of Neurology*.

[73] Winyard P. G., Blake D. R. (1996). Antioxidants, redox-regulated transcription factors, and inflammation. *Advances in Pharmacology*.

[74] Leppert D., Waubant E., Galardy R., Bunnett N. W., Hauser S. L. (1995). T cell gelatinases madiate basement membrane transmigration in vitro. *Journal of Immunology*.

[75] Karg E., Klivényi P., Németh I., Bencsik K., Pintér S., Vécsei L. (1999). Nonenzymatic antioxidants of blood in multiple sclerosis. *Journal of Neurology*.

[76] Vladimirova O., O'Connor J., Cahill A., Alder H., Butunoi C., Kalman B. (1998). Oxidative damage to DNA in plaques of MS brains. *Multiple Sclerosis*.

[77] Syburra C., Passi S. (1999). Oxidative stress in patients with multiple sclerosis. *Ukrainskii Biokhimicheskii Zhurnal*.

[78] Maccarrone M., Melino G., Finazzi-Agrò A. (2001). Lipoxygenases and their involvement in programmed cell death. *Cell Death & Differentiation*.

[79] Cooper R. L. T. (1997). Multiple sclerosis: an immune legacy?. *Medical Hypotheses*.

[80] Lehmann D., Karussis D., Misrachi-Koll R., Shezen E., Ovadia H., Abramsky O. (1994). Oral administration of the oxidant-scavenger N-acetyl-l-cysteine inhibits acute experimental autoimmune encephalomyelitis. *Journal of Neuroimmunology*.

[81] Malfroy B., Doctrow S. R., Orr P. L., Tocco G., Fedoseyeva E. V., Benichou G. (1997). Prevention and suppression of autoimmune encephalomyelitis by EUK-8, a synthetic catalytic scavenger of oxygen-reactive metabolites. *Cellular Immunology*.

[82] Hooper D. C., Bagasra O., Marini J. C. (1997). Prevention of experimental allergic encephalomyelitis by targeting nitric oxide and peroxynitrite: implications for the treatment of multiple sclerosis. *Proceedings of the National Academy of Sciences of the United States of America*.

[83] von Geldern G., Mowry E. M. (2012). The influence of nutritional factors on the prognosis of multiple sclerosis. *Nature Reviews Neurology*.

[84] Lovera J., Bagert B., Smoot K. (2007). Ginkgo biloba for the improvement of cognitive performance in multiple sclerosis: a randomized, placebo-controlled trial. *Multiple Sclerosis*.

[85] Wang Q., Chuikov S., Taitano S. (2015). Dimethyl fumarate protects neural stem/progenitor cells and neurons from oxidative damage through Nrf2-ERK1/2 MAPK pathway. *International Journal of Molecular Sciences*.

[86] Kawalec P., Mikrut A., Wisniewska N., Pilc A. (2014). The effectiveness of dimethyl fumarate monotherapy in the treatment of relapsing-remitting multiple sclerosis: a systematic review and meta-analysis. *Current Neuropharmacology*.

[87] Fox R. J., Miller D. H., Phillips J. T. (2012). Placebo-controlled phase 3 study of oral BG-12 or glatiramer in multiple sclerosis. *The New England Journal of Medicine*.

[88] Gold R., Kappos L., Arnold D. L. (2012). Placebo-controlled phase 3 study of oral BG-12 for relapsing multiple sclerosis. *The New England Journal of Medicine*.

[89] Phillips J. T., Fox R. J. (2013). BG-12 in multiple sclerosis. *Seminars in Neurology*.

[90] Fink J. K. (2013). Hereditary spastic paraplegia: clinico-pathologic features and emerging molecular mechanisms. *Acta Neuropathologica*.

[91] Blackstone C. (2012). Cellular pathways of hereditary spastic paraplegia. *Annual Review of Neuroscience*.

[92] Wakana Y., Koyama S., Nakajima K.-I. (2005). Reticulon 3 is involved in membrane trafficking between the endoplasmic reticulum and Golgi. *Biochemical and Biophysical Research Communications*.

[93] Orso G., Pendin D., Liu S. (2009). Homotypic fusion of ER membranes requires the dynamin-like GTPase atlastin. *Nature*.

[94] Lo Giudice T., Lombardi F., Santorelli F. M., Kawarai T., Orlacchio A. (2014). Hereditary spastic paraplegia: clinical-genetic characteristics and evolving molecular mechanisms. *Experimental Neurology*.

[95] Gücüyener K., Pinarli F. G., Erbaş D., Hasanoğlu A., Serdaroğlu A., Topaloğlu H. (2010). Is oxidative damage in operation in patients with hereditary spastic paraparesis?. *Brain and Development*.

[96] Casari G., De Fusco M., Ciarmatori S. (1998). Spastic paraplegia and OXPHOS impairment caused by mutations in paraplegin, a nuclear-encoded mitochondrial metalloprotease. *Cell*.

[97] Patel S., Latterich M. (1998). The AAA team: related ATPases with diverse functions. *Trends in Cell Biology*.

[98] McDermott C. J., Taylor R. W., Hayes C. (2003). Investigation of mitochondrial function in hereditary spastic paraparesis. *NeuroReport*.

[99] Ferreirinha F., Quattrini A., Pirozzi M. (2004). Axonal degeneration in paraplegin-deficient mice is associated with abnormal mitochondria and impairment of axonal transport. *The Journal of Clinical Investigation*.

[100] Atorino L., Silvestri L., Koppen M. (2003). Loss of m-AAA protease in mitochondria causes complex I deficiency and increased sensitivity to oxidative stress in hereditary spastic paraplegia. *The Journal of Cell Biology*.

[101] Milano A., Gesualdi N. M., Teperino R., Esposito F., Cocozza S., Ungaro P. (2005). Oxidative DNA damage and activation of c-Jun N-terminal kinase pathway in fibroblasts from patients with hereditary spastic paraplegia. *Cellular and Molecular Neurobiology*.

[102] Hansen J., Corydon T. J., Palmfeldt J. (2008). Decreased expression of the mitochondrial matrix proteases Lon and ClpP in cells from a patient with hereditary spastic paraplegia (SPG13). *Neuroscience*.

[103] Tesson C., Nawara M., Salih M. A. M. (2012). Alteration of fatty-acid-metabolizing enzymes affects mitochondrial form and function in hereditary spastic paraplegia. *American Journal of Human Genetics*.

